# Comparative Effectiveness and Safety of Rebamipide Versus Diquafosol in the Treatment of Dry Eye Disease: A Systematic Review and Meta-Analysis

**DOI:** 10.7759/cureus.116835

**Published:** 2026-09-24

**Authors:** Mehreen Iqbal, Huzaifa Rehman, Allah Rakhio Bhurgri, Daniel Abera, Shramik Rawal, Sonalben Chaudhary, Shamsha Hirani

**Affiliations:** 1 Ophthalmology, Rochdale Infirmary, Rochdale, GBR; 2 General Medicine, Avicenna Medical College, Lahore, PAK; 3 Opthalmology, Rochdale Infirmary, Rochdale, GBR; 4 Medicine, Dow University of Health Sciences, Karachi, PAK; 5 Ophthalmology, Bahir Dar University, Bahir Dar, ETH; 6 Internal Medicine, Mater Dei Hospital, Msida, MLT; 7 Internal Medicine, Zydus Sitapur Hospital, Sitapur, IND; 8 Cardiology, Baqai Hospital, Karachi, PAK

**Keywords:** diquafosol, dry eye disease, eye disease, rebamipide, systematic review and meta-analysis

## Abstract

This systematic review and meta-analysis aimed to compare the efficacy and tolerability of rebamipide (REB) and diquafosol (DQS), two widely used mucin secretagogues, in the treatment of dry eye disease. A comprehensive search of PubMed, Embase, Web of Science, Cochrane CENTRAL, and Scopus was conducted following Preferred Reporting Items for Systematic Reviews and Meta-Analyses (PRISMA) guidelines, identifying six comparative studies enrolling 277 patients across South Korea and Japan. Pooled analyses were performed using random-effects models, standardized mean differences (SMDs) for continuous outcomes and risk ratios (RRs) for dichotomous outcomes with 95% confidence intervals (CIs). Tear break-up time showed no significant difference between REB and DQS (SMD = -0.21 seconds, 95% CI: -0.52 to 0.11, p=0.20). Subjective symptom scores were also comparable (SMD=0.11, 95% CI: -0.16 to 0.38, p=0.40). Corneal staining scores were nearly identical (SMD=-0.002, 95% CI: -0.324 to 0.321, p=0.993), while conjunctival staining showed a nonsignificant trend favoring REB (SMD=-0.403, 95% CI: -1.508 to 0.702, p=0.475, I²=87.6%). These findings suggest comparable efficacy between both agents as first-line mucin secretagogues in the available studies, though formal equivalence cannot be established from this evidence base, and more large-scale trials are required to validate the findings.

## Introduction and background

Dry eye disease (DED) is a multifactorial disorder of the ocular surface characterized by tear film instability, hyperosmolarity, and chronic inflammation, and it remains one of the most common conditions encountered in ophthalmic practice worldwide [1,2]. According to the TFOS DEWS II framework, loss of tear film homeostasis triggers a self-perpetuating cycle of epithelial damage, goblet cell loss, and inflammatory cytokine release, which in turn further destabilizes the tear film [1]. Oxidative stress is now recognized as a key contributor to this cycle, as excessive reactive oxygen species accumulation promotes lipid peroxidation, mitochondrial dysfunction, and corneal epithelial apoptosis, thereby accelerating disease progression [3,4]. Given this pathophysiology, therapeutic strategies targeting tear film restoration and mucin secretion have become central to the staged management of DED, particularly for patients with short tear break-up time (TBUT)-type or aqueous-deficient subtypes who respond poorly to artificial tears alone [2,5].

Among the pharmacologic options available, diquafosol (DQS) and rebamipide (REB) are the two most widely prescribed topical mucin secretagogues, yet they act through fundamentally different mechanisms. DQS is a P2Y2 receptor agonist that stimulates fluid and mucin secretion from conjunctival epithelial and goblet cells via calcium-dependent chloride channel activation, thereby enhancing tear volume and lipid layer stability [6-8]. REB, originally developed as a gastric mucoprotective agent, promotes mucin-like glycoprotein synthesis through epidermal growth factor receptor signaling, increases goblet cell density, and exerts additional antioxidant and anti-inflammatory effects by suppressing reactive oxygen species generation and epithelial apoptosis [7,9]. These divergent mechanisms have prompted numerous head-to-head clinical comparisons across heterogeneous DED populations, including general dry eye cohorts [10], post-penetrating keratoplasty dry eye [11], Sjögren's syndrome-associated dry eye refractory to standard immunosuppressive therapy [12], post-refractive surgery dry eye [13], visual-display-terminal-associated dry eye among office workers [14], and, more recently, studies evaluating the immediate (30-minute) mucin-secreting and symptomatic effects of both agents [15].

Despite this expanding body of evidence, individual trials report inconsistent conclusions regarding the comparative efficacy of REB and DQS on core clinical outcomes-namely tear film stability, subjective symptom burden, ocular surface staining, and tolerability. Several studies report broadly comparable efficacy between the two agents [10,14,15], whereas others suggest a modest advantage of REB in reducing corneal and conjunctival staining and inflammatory markers in more severe, inflammation-predominant subtypes such as Sjögren's syndrome [9,12], or highlight tolerability trade-offs, including the bitter taste associated with REB versus greater patient-reported comfort with DQS [10,14]. These discrepancies likely stem from differences in study design (parallel-group, crossover, or paired-eye), underlying DED etiology, treatment duration, and heterogeneous outcome instruments, underscoring the need for a quantitative synthesis. To date, no meta-analysis has systematically pooled comparative data on REB versus DQS across these endpoints. This study therefore aimed to synthesize evidence evaluating the pooled effectiveness of REB versus DQS in DED.

## Review

Methodology

Literature Search and Search Strategy

A comprehensive literature search was conducted to identify studies comparing the therapeutic efficacy of REB and DQS in the management of DED. Electronic databases including PubMed/MEDLINE, Embase, Web of Science, Cochrane Central Register of Controlled Trials (CENTRAL), and Scopus were searched from inception to August 10, 2026, in accordance with the Preferred Reporting Items for Systematic Reviews and Meta-Analyses (PRISMA) guidelines [16]. The search strategy combined Medical Subject Headings (MeSH) terms and free-text keywords using Boolean operators, including combinations of "dry eye disease," "keratoconjunctivitis sicca," "rebamipide," "diquafosol," "mucin secretagogue," and "tear break-up time," A representative search string applied to PubMed was: ("dry eye"[MeSH] OR "keratoconjunctivitis sicca"[MeSH]) AND ("rebamipide"[tiab] OR "Mucosta"[tiab] OR "Rebaeye"[tiab]) AND ("diquafosol"[tiab] OR "Diquas"[tiab]). No language or publication date restrictions were applied at the initial search stage, and reference lists of retrieved articles and relevant prior reviews were manually screened to identify additional eligible studies not captured by the electronic search. Two reviewers independently screened titles and abstracts, followed by full-text assessment of potentially eligible articles, with disagreements resolved through discussion or consultation with a third reviewer.

Eligibility Criteria

Studies were included if they met the following PICOS criteria: (1) Population: patients clinically diagnosed with DED, irrespective of underlying etiology; (2) Intervention: topical REB ophthalmic suspension/solution; (3) Comparator: topical DQS ophthalmic solution; (4) Outcomes: at least one of the following: TBUT, subjective symptom scores (e.g., Ocular Surface Disease Index (OSDI), Dry Eye-Related Quality-of-Life Score (DEQS), corneal staining score, conjunctival staining score, or adverse events/tolerability; and (5) Study design: randomized controlled trials, prospective crossover trials, non-randomized studies, prospective or retrospective paired-eye comparative studies reporting extractable means, standard deviations, and sample sizes (or raw event counts for dichotomous outcomes) for both treatment arms. Studies lacking a direct REB-versus-DQS comparison (e.g., comparisons against sodium hyaluronate or artificial tears alone), animal or in vitro studies, case reports, conference abstracts without full data, and studies with non-extractable or duplicate data were excluded.

Data Extraction and Outcome Measures

Two reviewers independently extracted data using a standardized data-extraction form, including first author, publication year, country, study design, sample size, patient population/DED etiology, treatment regimen and duration, and outcome measures with corresponding mean ± standard deviation or event counts at the closest reported post-treatment time point. For studies with multiple follow-up visits, the primary post-treatment endpoint (or the longest available follow-up) was extracted as the main data point.

Risk of Bias Assessment

For quality assessment, the methodological quality of included studies was evaluated independently by two reviewers using tool appropriate to each study design. The five randomized, crossover, or paired-eye trials were assessed using the Cochrane Risk of Bias 2 (RoB 2) tool across five domains: randomization process, deviations from intended interventions, missing outcome data, measurement of the outcome, and selection of the reported result [17]. The single retrospective, nonrandomized comparative study was assessed separately using the Risk Of Bias In Non-randomized Studies of Interventions (ROBINS-I) tool, which evaluates bias across seven domains, including confounding and selection of participants, given its different design and susceptibility to bias relative to randomized trials [18]. Each domain was rated as low, some concerns/moderate, or high/serious risk of bias, and an overall judgment was assigned per study using the standard algorithms for each tool. Disagreements between reviewers were resolved by discussion.

Statistical Analysis

All statistical analyses were performed in Review Manager (RevMan) Version 5.4 (The Cochrane Collaboration, Copenhagen, Denmark). For continuous outcomes reported on the same measurement scale across studies (TBUT, in seconds), the mean difference (MD) with 95% confidence interval (CI) was used as the summary effect measure. For outcomes assessed with heterogeneous instruments or scoring systems (subjective symptom scores, corneal staining scores, conjunctival staining scores), the standardized mean difference (SMD) was used. For dichotomous outcomes (adverse events/tolerability), the risk ratio (RR) with 95% CI was calculated using the Mantel-Haenszel method. All pooled estimates were derived using a random-effects model method of estimating between-study variance (τ²), given the anticipated clinical and methodological heterogeneity across included studies (varying DED etiologies, treatment durations, and study designs) [19]. Statistical heterogeneity was assessed using the Cochran Q test and the I² statistic, with I² values of 25%, 50%, and 75% considered to represent low, moderate, and high heterogeneity, respectively [20,21]. Results were visualized using forest plots displaying individual study estimates, pooled effect sizes, and 95% CIs, with REB designated as the reference (experimental) group and DQS as the comparator throughout, such that effect estimates favoring REB were represented as negative values for symptom and staining outcomes and positive values for TBUT. Publication bias was assessed using Funnel plot. As number of studies assessing each outcome was less than 10, formal statistical assessment of publication bias was not possible. A two-sided p-value <0.05 was considered statistically significant for all pooled estimates.

Results

Figure 1 presents the study selection process. The initial database search identified 483 records. After removal of duplicates, 436 records were screened based on their titles and abstracts. Following the initial screening, the full texts of 14 potentially eligible studies were retrieved and assessed for eligibility. After a detailed full-text assessment, six studies met the inclusion criteria and were included in the meta-analysis. The included studies were conducted across South Korea, Japan, and involved a range of study designs. Follow-up duration ranged from 30 minutes to three months. Underlying DED etiologies varied across studies and included general/idiopathic dry eye, post-penetrating keratoplasty dry eye, and refractory Sjögren's syndrome-associated dry eye, reflecting the clinical heterogeneity typical of comparative DED trials. Detailed characteristics of included studies are presented in Table 1. Table 2 and Table 3 present risk of bias assessment of included studies.

**Figure 1:**
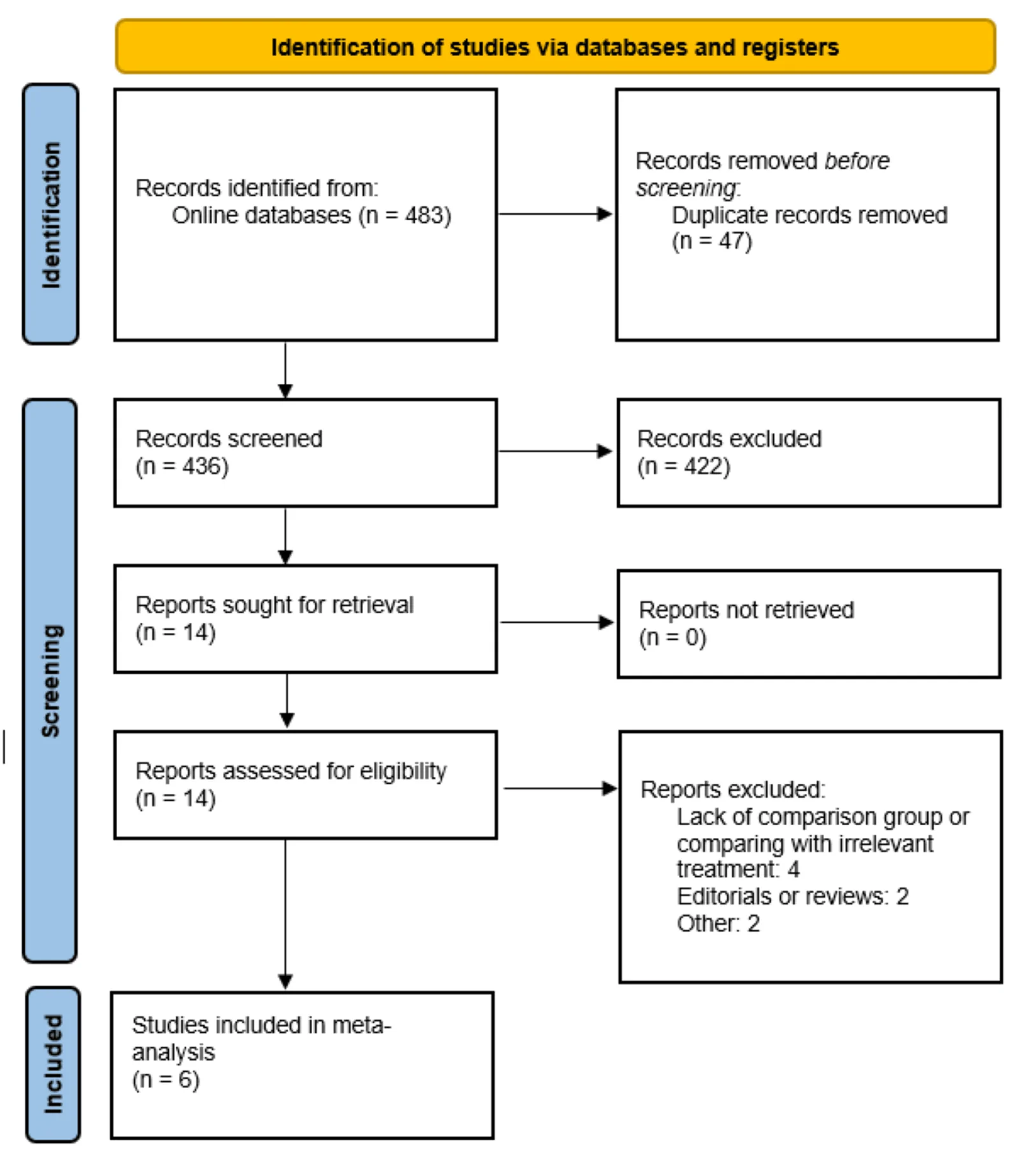
PRISMA flowchart (study selection process) PRISMA: Preferred Reporting Items for Systematic Reviews and Meta-Analyses

**Table 1:** Characteristics of included studies

Author (Year)	Country	Study Design	Sample Size (N)	Follow-up	Mean Age (Years)	Male, n (%)
Hwang et al. (2026) [22]	South Korea	Retrospective, nonrandomized comparative observational study	60 patients (120 eyes)	3 months	59	12 (20.0%)
Jin et al. (2024) [10]	South Korea	Prospective randomized crossover trial	28 patients (28 eyes)	10 weeks	61.3	8 (28.6%)
Kobashi et al. (2017) [11]	Japan	Prospective randomized parallel-group trial	40 patients (40 eyes)	4 weeks	70	21 (52.5%)
Lee et al. (2025) [12]	South Korea	Prospective randomized parallel-group trial	52 patients (52 eyes)	3 months	60	1 (1.9%)
Shimazaki et al. (2017) [14]	Japan	Prospective randomized multicenter parallel-group trial	67 patients (67 eyes)	4 weeks	40	20 (29.9%)
Song et al. (2026) [15]	South Korea	Prospective randomized double-blinded paired-eye trial	30 patients (60 eyes)	30 minutes	34.8	7 (23.3%)

**Table 2:** Risk of bias assessment of included RCTs using Cochrane risk of bias assessment-2 D1, bias arising from the randomization process; D2, bias due to deviations from intended interventions; D3, bias due to missing outcome data; D4, bias in measurement of the outcome; D5, bias in selection of the reported result. RCTs: Randomized controlled trials

Study (Year)	D1	D2	D3	D4	D5	Overall Risk of Bias
Jin et al. (2024) [10]	Low	Some concerns	Some concerns	Low	Low	Some concerns
Kobashi et al. (2017) [11]	Some concerns	Low	Low	Some concerns	Low	Some concerns
Lee et al. (2025) [12]	Some concerns	Low	Low	Some concerns	Low	Some concerns
Shimazaki et al. (2017) [14]	Some concerns	Some concerns	Low	High	Low	High
Song et al. (2026) [15]	Low	Low	Low	Some concerns	Low	Some concerns

**Table 3:** Risk of bias assessment of the non-RCT D1, bias due to confounding; D2, bias in selection of participants into the study; D3, bias in classification of interventions; D4, bias due to deviations from intended interventions; D5, bias due to missing data; D6, bias in measurement of outcomes; D7, bias in selection of the reported result. RCT: Randomized controlled trial

Study (Year)	D1. Confounding	D2. Selection of Participants	D3. Classification of Interventions	D4. Deviations from Intended Interventions	D5. Missing Data	D6. Measurement of Outcomes	D7. Selection of Reported Result	Overall Risk of Bias
Hwang et al. (2026) [22]	Serious	Moderate	Low	Moderate	Moderate	Moderate	Low	Serious

Meta-analysis of outcomes

Tear Break-up Time

Figure 2 presents pooled analysis comparing TBUT between DQS and REB in patients with DED. Pooled analysis across all five studies (k=5) demonstrated no statistically significant difference between REB and DQS in TBUT (SMD = -0.21 seconds, 95% CI: -0.52 to 0.11, p=0.20), with no observed between-study heterogeneity (I² = 0%, p-value=0.98). The funnel plot for TBUT includes only five studies (figure 3), showing a roughly symmetric scatter around the SMD reference line, with no strong visual evidence of publication bias. However, given the very small number of studies, funnel plot asymmetry cannot be reliably assessed, and results should be interpreted with caution.

**Figure 2:**
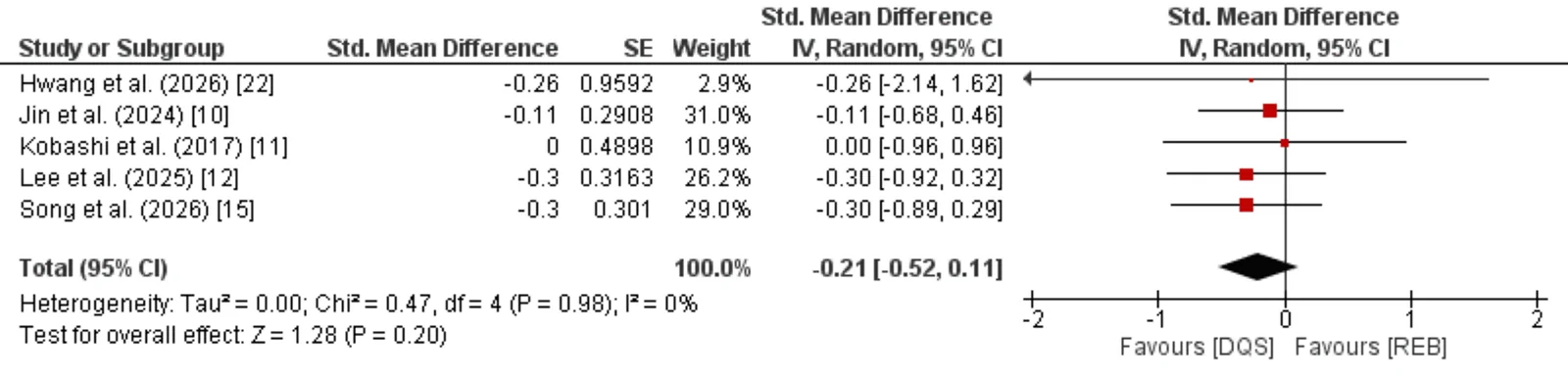
Forest plot comparing TBUT References [10-12,15,22] Individual study estimates (red squares, sized proportional to the inverse-variance weight assigned in the random-effects model) and their 95% confidence intervals (CIs, horizontal lines) are shown for each of the five included studies, together with the pooled summary estimate (black diamond, width representing its 95% CI). Values greater than zero favor REB (longer, more stable TBUT), and values less than zero favor DQS. REB: Rebamipide; DQS: diquafosol; TBUT: tear break-up time

**Figure 3:**
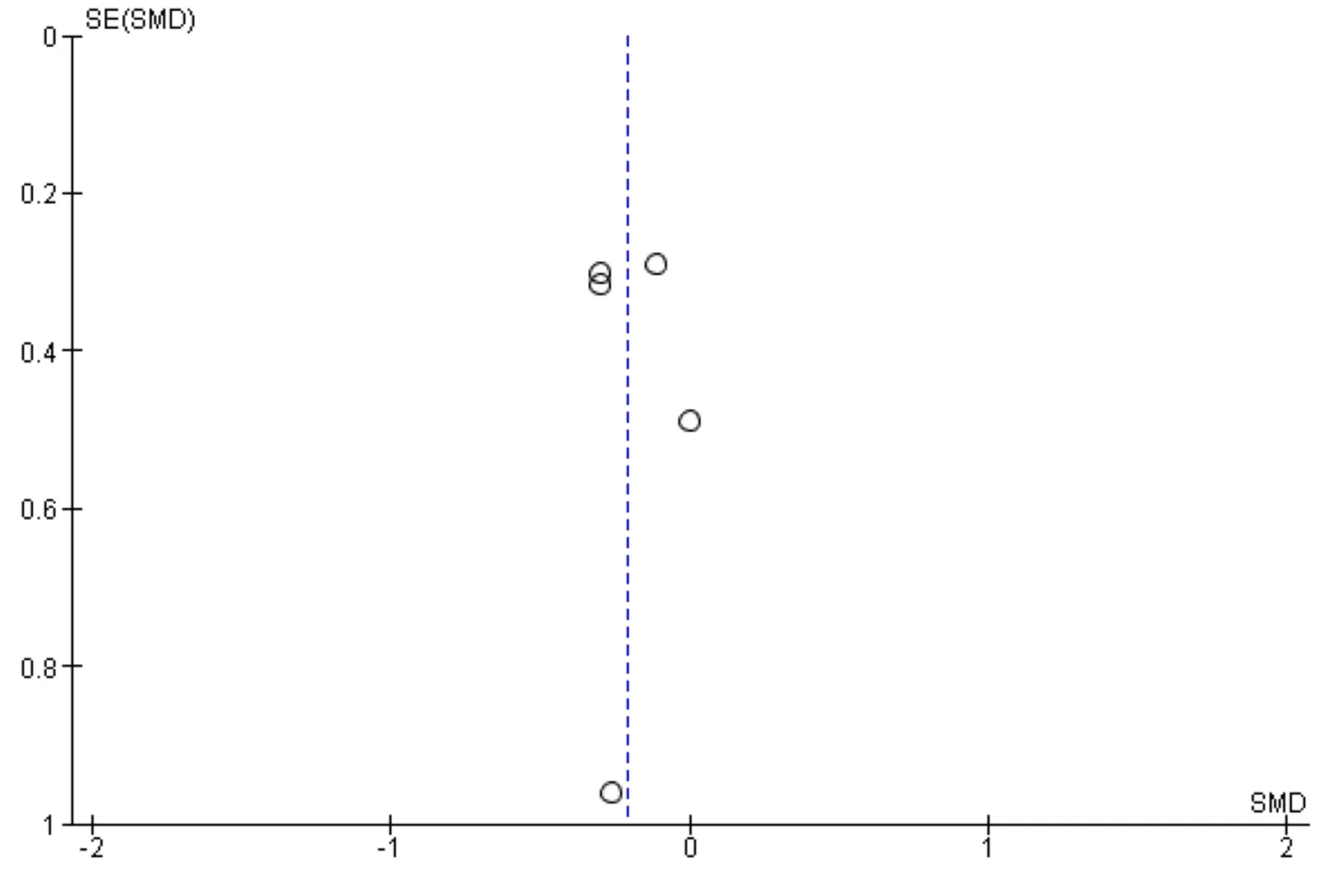
Funnel plot of the TBUT outcome (publication bias) TBUT: Tear break-up time; SMD: standardized mean difference

Subjective Symptom Score

Figure 4 presents pooled analysis comparing subjective symptom score between DQS and REB in DED. The pooled SMD was 0.11 (95% CI: -0.16 to 0.38, p=0.40), with no heterogeneity (I² = 0%, Q p=0.40), indicating no significant difference in subjective symptomatic relief between REB and DQS. The funnel plot for Subjective Symptom Score includes four studies (Figure 5), clustered closely with similar precision (SE ≈ 0.15-0.30) and roughly symmetric distribution around the SMD reference line. This suggests no obvious publication bias, though with only four studies, formal assessment of asymmetry remains statistically unreliable.

**Figure 4:**
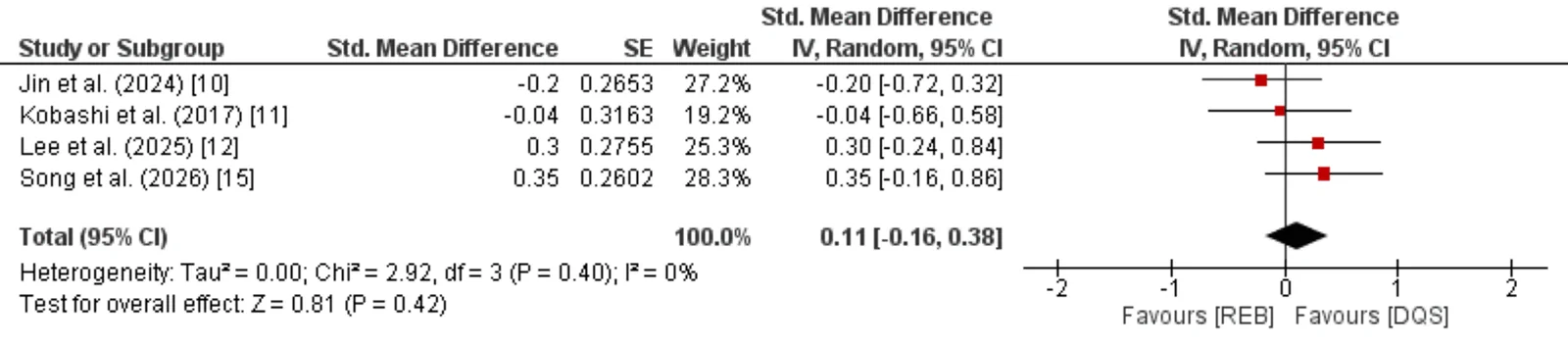
Forest plot comparing the subjective symptom score References [10-12,15] Individual study estimates (red squares, sized proportional to the inverse-variance weight assigned in the random-effects model) and their 95% confidence intervals (CIs, horizontal lines) are shown for each of the five included studies, together with the pooled summary estimate (black diamond, width representing its 95% CI). Values greater than zero favor DQS and values less than zero favor REB. REB: Rebamipide; DQS: diquafosol; TBUT: tear break-up time

**Figure 5:**
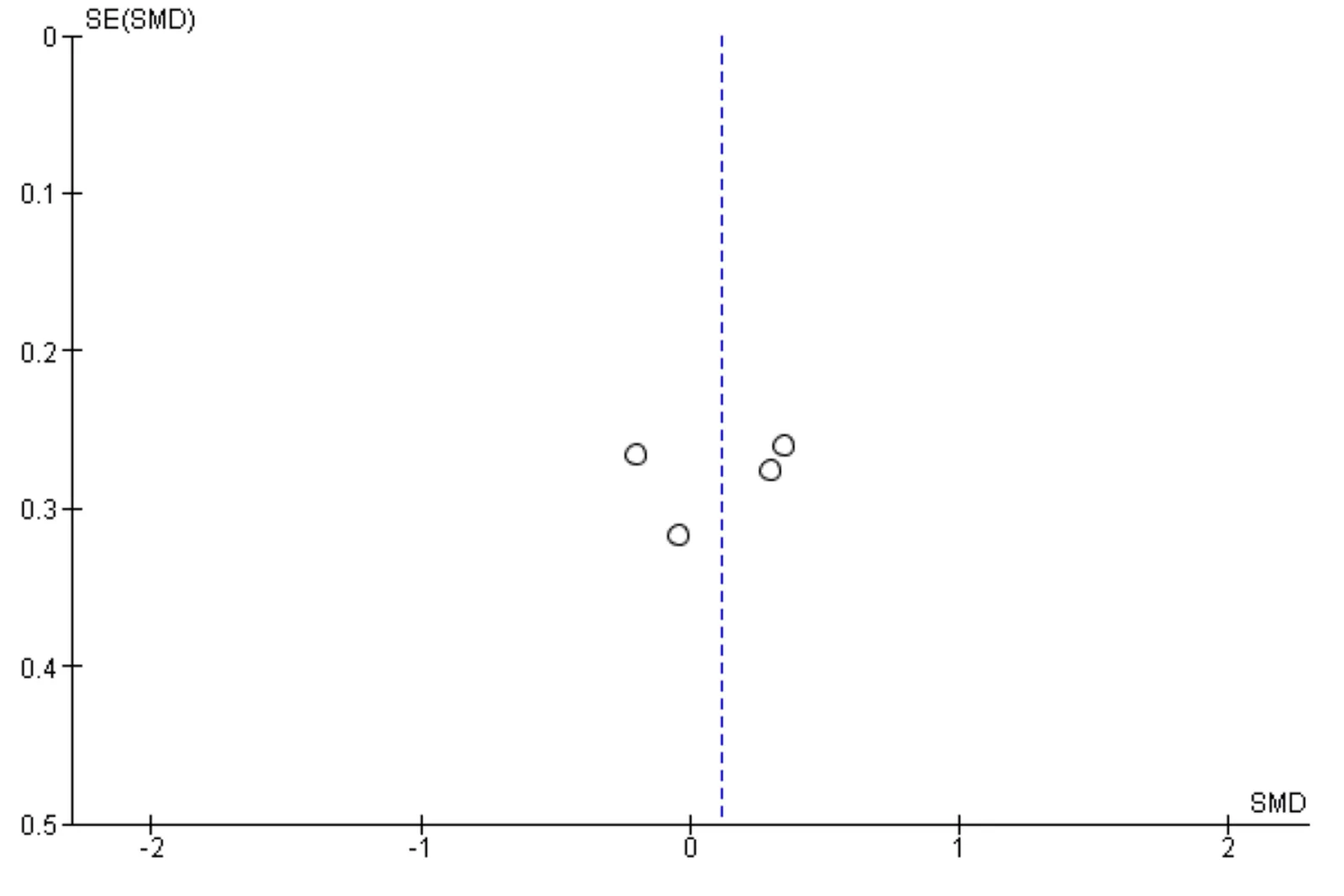
Funnel plot of the subjective symptom score outcome (publication bias) SMD: Standardized mean difference

Sensitivity Analysis

To assess the robustness of the primary pooled estimates to two major sources of methodological heterogeneity, within-subject correlation in the crossover-design study and the inclusion of an immediate (30-minute) pharmacodynamic assessment, sensitivity analyses were performed for the TBUT and subjective symptom score outcomes.

Excluding Jin et al. (2024) [10], the sole crossover-design study, the pooled estimate for TBUT was materially unchanged (SMD = -0.25, 95% CI: -0.63 to 0.13, p=0.20, I²=0%, k=4) relative to the primary analysis (SMD = -0.21, 95% CI: -0.52 to 0.11, p=0.20, k=5). For the subjective symptom score, exclusion of Jin et al. shifted the pooled estimate modestly toward favoring REB (SMD = 0.23, 95% CI: -0.09 to 0.55, p=0.16, I²=0%, k=3) compared with the primary estimate (SMD = 0.11, 95% CI: -0.16 to 0.38, p=0.40, k=4); statistical significance and low heterogeneity were preserved in both analyses.

Excluding Song et al. (2026) [15], the study with a 30-minute follow-up, the pooled estimate for TBUT likewise remained nonsignificant and similar in magnitude (SMD = -0.17, 95% CI: -0.55 to 0.21, p=0.38, I²=0%, k=4). For the subjective symptom score, exclusion of Song et al. shifted the pooled estimate close to the null (SMD = 0.02, 95% CI: -0.30 to 0.34, p=0.91, I²=0%, k=3), suggesting that this acute-timepoint study contributed modestly to the direction of the primary estimate without altering its nonsignificance.

Secondary Outcomes

Table 4 presents pooled analysis of secondary outcomes. Corneal staining scores, pooled as SMD across three studies, showed no significant difference between REB and DQS. Conjunctival staining scores, pooled across two studies, showed a nonsignificant trend favoring REB. However, this estimate warrants cautious interpretation given the small number of contributing studies and substantial heterogeneity (I² = 87.6%). Dichotomous tolerability outcomes, each pooled across two studies, showed no significant between-group differences in the risk of foreign body sensation or stinging/burning discomfort.

**Table 4:** Secondary outcomes Corneal and conjunctival staining scores were pooled as the standardized mean difference (SMD) because the contributing studies used different staining instruments. Foreign body sensation and stinging/burning discomfort were pooled as the risk ratio (RR) using the Mantel–Haenszel method for dichotomous data. REB was designated the reference (numerator) group and DQS the comparator throughout; therefore, negative SMD values and RR<1 favor REB, whereas positive SMD values and RR>1 favor DQS. SMD: Standardized mean difference; RR: risk ratio; LCI: lower confidence interval; UCI: upper confidence interval; REB: Rebamipide; DQS: diquafosol

Outcome	Estimate	LCI	UCI	P-Value	I-Square
Corneal staining (SMD)	-0.002	-0.324	0.321	0.9926	0
Conjunctival staining (SMD)	-0.403	-1.508	0.702	0.4752	87.6
Foreign body sensation (RR)	0.73	0.368	1.449	0.3684	0
Stinging/burning (RR)	0.689	0.346	1.369	0.2874	0

Discussion

In this systematic review and meta-analysis of six comparative studies enrolling 277 patients with DED, we evaluated the comparative efficacy and tolerability of REB and DQS, two structurally and mechanistically distinct mucin secretagogues that are used interchangeably in tear film-oriented therapy. Overall, our pooled findings indicate that REB and DQS produce broadly comparable improvements in tear film stability, subjective symptom burden, and ocular surface staining, with no statistically significant between-group differences identified across the efficacy and safety outcomes.

TBUT, widely regarded as the most clinically relevant indicator of tear film stability and a cornerstone diagnostic criterion in both the TFOS DEWS II and Asia Dry Eye Society frameworks [1,2], showed no meaningful between-group difference. This finding is mechanistically plausible: although DQS and REB act through distinct receptor pathways; DQS as a P2Y2 receptor agonist stimulating calcium-dependent chloride channel-mediated fluid and mucin secretion, and REB via epidermal growth factor receptor-mediated upregulation of membrane-bound and secretory mucins [6,7]. Both drugs ultimately converge on restoring the mucin-dependent wettability of the corneal epithelium that underlies tear film breakup. Because short-BUT phenotype dry eye is thought to arise primarily from a qualitative mucin deficiency rather than volumetric tear insufficiency [23], the equivalence observed here suggests that either agent's shared mucin-secretagogue action is sufficient to restore tear film stability, regardless of whether aqueous secretion is additionally augmented.

Pooled subjective symptom showed no significant difference between agents. Symptom relief in DED is multifactorial, reflecting reduced tear hyperosmolarity, inflammatory burden, and lid-wiper friction rather than a single pharmacological action [1,3,24]. Both drugs enhance mucin-dependent lubrication: DQS via increased aqueous volume, and REB via mucin upregulation plus antioxidant and anti-inflammatory effects limiting epithelial apoptosis [7,9], suggesting convergent symptomatic relief despite differing mechanisms. Clinically, this equivalence suggests patient-reported outcomes are unlikely to meaningfully differentiate the two drugs for treatment selection. Corneal staining scores were essentially identical, while conjunctival staining showed a nonsignificant trend favoring REB (SMD = -0.403), driven largely by one study in refractory Sjögren's-associated dry eye [12], where REB's antioxidant and anti-inflammatory properties may benefit more severe, inflammation-predominant phenotypes [3,4,9]. However, as this signal derived from only two studies with discordant estimates and substantial heterogeneity (I² = 87.6%), it should be considered hypothesis-generating, warranting prospective replication in inflammation-predominant DED subtypes.

Our findings are broadly consistent with prior head-to-head trials directly comparing these two agents, which have similarly reported comparable TBUT and symptom-score outcomes between REB and DQS [10,14]. They also align with recent single-agent meta-analyses confirming REB's efficacy relative to artificial tears across TBUT, staining, and Schirmer outcomes [25], supporting the biological plausibility of therapeutic equivalence between the two secretagogues observed in our comparative synthesis. Nonetheless, the substantial heterogeneity observed in the conjunctival staining estimate (I² = 87.6%) should not be dismissed as a statistical artifact. Plausible contributors include differences in baseline disease severity and underlying etiology (idiopathic versus Sjögren's syndrome-associated dry eye), differing diagnostic criteria (short-BUT phenotype versus broader NEI/DEWS-based definitions), variable treatment duration (30 minutes to three months) and dosing frequency, heterogeneous staining grading systems (NEI, SICCA, Oxford, and fluorescein 0-9 scales), and differing study designs (prospective randomized versus retrospective observational), all of which limit the precision and generalizability of this specific pooled estimate and underscore the need for standardized outcome reporting in future comparative DED trials.

Although pooled dichotomous analyses of foreign body sensation and stinging/burning discomfort showed no significant between-group differences, bitter taste emerged as a notable REB-specific complaint. In one trial, bitter taste was reported as a treatment-emergent adverse event in 100% of REB-treated eyes [10], a finding derived from a single study and requiring cautious interpretation given the absence of corroborating data from other included trials. Separately, in the retrospective cohort by Hwang et al., poor taste was cited as the leading reason for treatment discontinuation, affecting 20.0% of REB-treated patients [22], compared with ocular discomfort as the primary reason for DQS discontinuation in 10.0% of patients; this reflects discontinuation data rather than treatment-emergent adverse event frequency, and the two outcome types should not be conflated. This taste-related tolerability signal most likely reflects the drug's gustatory profile following nasolacrimal duct drainage rather than any ocular toxicity signal and newer REB clear-solution formulations incorporating mannitol, arginine, and menthol have been specifically engineered to mitigate this problem, with early comparative data suggesting reduced bitterness relative to the original suspension formulation [22,26]. Nonetheless, because poor tolerability is a well-established driver of non-adherence in chronic topical DED therapy, this finding carries direct clinical weight even in the absence of any difference in objective efficacy measures.

Several limitations warrant consideration. First, the number of included studies contributing to each outcome was small (k=2-5), limiting statistical power to detect modest between-group differences and precluding meaningful assessment of publication bias, such as Egger's regression test for funnel plot asymmetry. Second, follow-up duration varied nearly 150-fold across studies (30 minutes to three months), meaning pooled estimates conflate acute pharmacodynamic effects with cumulative therapeutic benefit. Third, dosing frequency differed between agents in several studies (DQS typically administered six times daily versus REB four times daily), so observed between-group differences, or the lack thereof, cannot be fully disentangled from administration frequency rather than intrinsic pharmacologic effect. Fourth, all included studies originated from East Asian populations (South Korea and Japan), where both agents are widely available and short-BUT phenotype dry eye is more frequently diagnosed and where DED prevalence and phenotype distribution may differ from Western cohorts, limiting generalizability to populations where these secretagogues remain less accessible. Additionally, the pooled populations in this review span clinically and pathophysiologically distinct DED etiologies, including general/idiopathic, post-penetrating keratoplasty, and refractory Sjögren's syndrome-associated disease, which differ in inflammatory burden and expected treatment response. Given the limited number of studies representing each etiology, formal subgroup analysis was not feasible, and pooled estimates should be interpreted as an average treatment effect across heterogeneous populations rather than evidence of a uniform effect across DED phenotypes.

Future research should prioritize adequately powered, prospective randomized trials with matched dosing frequency and standardized outcome instruments, particularly a single validated staining scale across defined DED subtypes; extended follow-up beyond three months to capture cumulative treatment effects; and combination-therapy trials exploring whether the complementary mechanisms of these two secretagogues confer additive benefit when co-administered, an approach not yet formally evaluated in the current evidence base.

## Conclusions

REB and DQS showed comparable efficacy across tear film stability and ocular surface staining in patients with DED, with no statistically significant differences observed between the two mucin secretagogues on these outcomes. For subjective symptom relief, pooled estimates likewise showed no statistically significant difference; however, given the limited number of contributing studies and the heterogeneity of symptom assessment instruments, baseline severity, and follow-up duration across these trials, the available evidence may be insufficient to exclude clinically meaningful differences in patient-reported outcomes, and this finding should be interpreted with corresponding caution. These findings suggest comparable efficacy between both agents as first-line mucin secretagogues in the available studies, though formal equivalence cannot be established from this evidence base. Larger, adequately powered prospective trials with standardized outcome measures and matched dosing regimens are warranted to confirm these findings, particularly for patient-reported symptom outcomes.
